# Repurposing FDA-Approved Drugs as Hendra Virus RNA-Dependent RNA Polymerase Inhibitors: A Comprehensive Computational Drug Discovery Approach

**DOI:** 10.3390/v17121613

**Published:** 2025-12-13

**Authors:** Anjana C. Lalu, Varun Thachan Kundil, Bristow Ben Joseph, Radul R. Dev, Amritha Thaikkad, Suhail Subair, Rajesh Raju, Abhithaj Jayanandan

**Affiliations:** Centre for Integrative Omics Data Science (CIODS), Yenepoya (Deemed to be University), Mangaluru 575018, Karnataka, India; anjanaclalu63@gmail.com (A.C.L.); varuntk.me3@gmail.com (V.T.K.); bristowben987@gmail.com (B.B.J.); radulrdev@gmail.com (R.R.D.); amrithat.ciods@yenepoya.edu.in (A.T.); suhailsubair.ciods@yenepoya.edu.in (S.S.)

**Keywords:** hendra virus, RNA-dependent RNA polymerase, FDA-approved drugs, molecular dynamics

## Abstract

Hendra virus (HeV) is a highly pathogenic zoonotic paramyxovirus that poses a serious threat to human and equine health, yet no approved antivirals or vaccines currently exist. RNA-dependent RNA polymerase (RdRp) of Hendra virus represents a critical and attractive target for antiviral drug development, given its essential role in both viral genome replication and mRNA transcription. Due to the lack of a human homolog, it is more druggable and less likely to cause host toxicity. Its sequence conservation among related paramyxoviruses further highlights its potential for the development of broad-spectrum inhibitors. This study offers the first comprehensive computational analysis of the Hendra virus RdRp, potentially promising FDA-approved drugs as possible inhibitors. A homology model of RdRp was generated in the absence of experimental three-dimensional (3D) structure, followed by virtual screening and molecular dynamics (MD) simulations to evaluate the drug binding and stability. Based on the highest energy, four FDA-approved drugs selected were menadiol diphosphate (−49.88 kcal/mol), masoprocol (−39.69 kcal/mol), pamidronic acid (−34.29 kcal/mol), and dinoprostone (−46.90 kcal/mol). Furthermore, these compounds exhibited significant interactions with the catalytic GDNE motif. With strong conformational stability and pharmacokinetic profile, masoprocol and menadiol diphosphate showed the most stable and energetically favorable interactions within the RdRp active site. These findings suggest their potential as repurposed therapeutic candidates against Hendra virus infection and they provide a structural basis for the development of broad-spectrum paramyxovirus inhibitors, justifying additional experimental confirmation.

## 1. Introduction

Human infection with the Hendra virus (HeV) is a zoonotic illness with a low infectivity and a high fatality rate. Through an intermediate equine host, the illness has been spread from bats of the genus Pteropus (flying foxes) to humans [1]. Hendra and Nipah viruses are members of the genus Henipavirus in the family Paramyxoviridae, subfamily Paramyxovirinae. HeV has been a major public health concern since its initial outbreak in the Australian suburb of Hendra, close to Brisbane, in 1994. All human cases that were reported have been fatal. HeV-g2, a novel genotype discovered in 2021 close to Newcastle, Australia, highlights the virus’s continuous development and potential for spillover [2]. High rectal temperatures, acute respiratory distress, and influenza-like symptoms, such as nasal discharge, were the hallmarks of the sickness [3,4].

HeV enters host cells through the combined action of the attachment (G) and fusion (F) glycoproteins, which enable the viral fusion and the release of RNA into the host cytoplasm. The RNA-dependent RNA polymerase (RdRp), which is made up of the large (L) and phosphoprotein (P) subunits, starts the transcription of viral RNA into mRNAs for the production of viral proteins. The RdRp switches from transcription to replication when there is enough nucleocapsid (N) protein available, producing full-length RNA copies for new viral particles. The NiV L RdRp domain has seven catalytic motifs (A–G), including the essential catalytic “GDNE” motif (Gly831–Glu834) [5]. For therapeutic interventions the conserved GDNE (831–834) motif of the RdRp domain is relevant, which forms the catalytic core of the polymerase [6].

Currently, there are no approved vaccinations or antiviral medications available for human use [7,8]. The high fatality rate and the emergence of new genotypes underscore the necessity for novel and effective therapeutics. Molecular docking and other structure-based drug discovery techniques provide useful tools for identifying possible inhibitors that target important viral proteins [9]. Successful examples include Probenecid has been repurposed to prevent hMPV [10,11,12]. Additionally, remdesivir has been shown to exhibit antiviral activity against the Hendra virus [13].

Despite the high genetic resemblance between the Hendra and Nipah virus, Hendra virus has unique evolutionary and epidemiological characteristics, including recurrent spillover events in Australia and the emergence of HeV-g2, which emphasizes the urgent need for the effective treatment strategies. Furthermore, minor variations within the catalytic domain between Hendra and Nipah virus may affect drug binding affinity, indicating the importance of studying Hendra RdRp independently. In order to address HeV and its developing variations, this integrative method aims to speed up the development of promising therapy candidates.

## 2. Methodology

As there is no crystal structure available for the RdRp protein of the Hendra virus, modeling approaches were used. Schrödinger Maestro is employed to identify the protein–ligand interactions and the binding affinity of FDA-approved drugs with the target protein. Molecular docking analysis is performed to predict the binding affinities and interactions between ligands and targets. Molecular dynamics simulations were carried out to assess the dynamics behavior and stability. Molecular dynamics simulations were used to assess the ligand target complex’s stability and dynamic behavior.

### 2.1. Homology Modeling

In the absence of an experimentally determined crystal structure, the homology modeling approach enables the development of viable models by superimposing the experimentally determined structure of a closely related protein (referred to as the “template”) with the amino acid sequence of the protein under prediction (referred to as the “target”) [14]. Before the query sequence is mapped onto the template’s framework, possible template candidates are identified using sequence alignment. The target and template must have a high degree (86.72%) of sequence similarity for the model to be accurate. To construct the 3D structure of HeV RdRp, the template was identified using the amino acid sequence of the RdRp protein of the Hendra virus as a query sequence (UniProt ID: O89344, corresponding to the L polymerase domain of Hendra virus). Potential templates have been identified from the SWISS-MODEL Template Library (SMTL) using the BLAST 2.17.0. Following that, the Quaternary Structure Quality Estimate (QSQE) score was used to rank these templates. The PDB entry 9BDQ, which denotes a biological assembly (quaternary structure) rather than a monomeric chain, is the template used for this study. The SWISS-MODEL server prioritizes and provides the Quaternary Structure Quality Estimate (QSQE) metric rather than GMQE for such oligomeric templates, as QSQE has been specifically designed to evaluate the accuracy and confidence of predicted quaternary interfaces. Furthermore, the server did not create QMEAN for this modeling case, which frequently happens when oligomeric templates are utilized. As a result, QSQE was chosen as the most suitable and accessible quality indicator for this study’s model evaluation. The best template was selected, and the variable parts, such as insertions or deletions, were improved by loop modeling, while conserved areas were extracted directly from the template [15]. Following the optimization and validation, the final homology model was then used for the downstream molecular docking and molecular dynamics simulations.

### 2.2. Protein Preparation

Using the Protein Preparation Wizard of Schrodinger Suite (version 2024-3), the homology-modeled structure of the target protein was preprocessed [16,17]. In the preprocessing steps, the metal ions, hetero groups, and water molecules located beyond 5 Å were removed, and the disulfide bonds, bond orders, and formal charges were assigned. Hydrogen atoms were added to stabilize the structure and the PROPKA module was used to identify the protonation states of ionizable residues at a physiological pH of 7.4 ± 2.0. The energy of the structure is minimized using the OPLS4 force field. OPLS4 leads to improved accuracy on benchmarks that assess small-molecule solvation and protein–ligand binding [18]. In order to refine the hydrogen atom placement, the Impref utility was used to optimize the positioning of the heavy atoms. The “H-bond assignment” tool was used to adjust the hydrogen-bonding network.

### 2.3. Ligand Preparation

The 3D structure of 2449 FDA-approved drugs was retrieved from DrugBank [19]. All the compounds were preprocessed using the LigPrep module of the Schrodinger suite. The Epik Classic module was used to create potential ionization and tautomeric states at a target pH of 7.0 ± 2.0 which represents physiological conditions. All possible tautomers were generated and chiralities were determined from the 3D structures. To ensure that all ligands were adequately prepared for ensuing molecular docking and simulations, the OPLS_4 force field was utilized to optimize the molecular geometry and refine atomic charges to find the ideal ligand conformations.

### 2.4. Receptor Grid Generation and Virtual Screening Using Molecular Docking

Using the “receptor grid generation” module, a grid of 10 × 10 × 10 Å was generated. Potential binding sites on the RdRp of the Hendra virus were explored and characterized using the default settings in Schrödinger’s SiteMap. SiteMap searches the complete protein to identify potential locations for ligand binding and assesses the druggability of these sites, typically when the location of binding sites is unknown [20]. In virtual screening, potential binding positions will be predicted by analyzing the interaction between a ligand and a target. Docking assesses each ligand molecule’s fit into a protein binding site by a method that often involves evaluating hundreds of possible configurations. Each pose is then evaluated for fit using various scoring methods. The GLIDE module was used to develop a virtual screening process. This module uses grid-based ligand docking to estimate ligand-binding poses and affinities [21,22]. The virtual screening methodology of the Glide module allows the thorough analysis of the top hits on the phase screen [23]. This process includes high-throughput virtual screening (HTVS), standard precision (SP), and extra precision (XP) docking. After every docking stage, the lead compounds were filtered, and the compounds with the highest docking scores—the top 30%—were chosen for further study. The binding free energy was also computed using the Prime module of the Schrödinger suite’s MM-GBSA (Molecular Mechanics Generalized Born Surface Area) in accordance with the XP docking data. Based on their binding free energy and docking scores, the top compounds were chosen.

### 2.5. ADMET Analysis

Using in silico ADMET (absorption, distribution, metabolism, excretion, and toxicity) prediction methods, the toxicity and pharmacokinetic properties of the selected drugs were evaluated. ADMET analysis is essential to drug research and development. In addition to reducing all of the costs and time related to experimental testing, this method allows for the early evaluation of drug likeness and safety. When assessing drug likeness, established criteria like Lipinski’s rule-of-five and physicochemical properties like molecular weight, hydrogen-bond donors and acceptors, lipophilicity, and oral bioavailability were taken into consideration. Many clinical trials fail because of the lack of ADMET characteristics [24]. To examine the pharmacokinetic and pharmacodynamic characteristics of drug leads, we collected SMILES from the PubChem databases using AI Drug Lab [25]. These computational analyses were used to systematically evaluate every compound across multiple ADMET categories. First, unsuitable compounds were eliminated by screening using physicochemical and drug-likeness criteria (Lipinski’s rule of five, solubility, and lipophilicity). Then, to determine the potential for absorption and distribution, important pharmacokinetic properties such oral bioavailability, blood–brain barrier permeability, and plasma membrane binding were examined. Finally, toxicity-related factors such as acute toxicity, drug-induced liver damage, and Ames mutagenicity were evaluated in order to screen out high-risk candidates. Compounds were found to have favorable safety and effectiveness profiles and were thus shortlisted to be potential therapeutic candidates for further investigation if they satisfied the acceptable criteria across these combined parameters.

### 2.6. Molecular Dynamic Simulation

Molecular Dynamics (MD) simulations were used to analyze the dynamic stability and flexibility of protein–ligand complexes. In the protein–ligand docked complex, MD provides structural integrity and conformational changes by computing the physical movement of atoms and molecules [26]. Following virtual screening, the most promising compounds were selected and their stability was evaluated using MD simulations. The system was built by placing a protein–ligand complex in a water box made of TIP3P followed by neutralization by adding Na⁺ and Cl^−^ by replacing water molecules. Periodic boundary conditions were applied in all directions, and a cutoff of 9.0 Å was used for short-range van der Waals and electrostatic interactions. The system was equilibrated in both NVT and NPT ensembles using a Berendsen thermostat and barostat (at 10 K for 12 ps and 300 K for 24 ps) after a multi-step relaxation approach that included both restricted and unrestrained energy minimization. The MD simulation ran consistently for 200 ns via NPT ensemble at 300 K and 1.013 bar of pressure using a Nose–Hoover thermostat and a Martyna–Tobias–Klein barostat with a 2 fs time step [27]. The OPLS4 force field was used for energy minimization and during the simulations, 2000 frames were recorded for each system. After the simulation, we used the thermal_mmgbsa.py script to compute the binding free energies using the MM-GBSA method on 21 frames extracted at 50-frame intervals from the last 100 ns of the 200 ns MD trajectory. The root mean square deviation (RMSD), root mean square fluctuation (RMSF), ligand interactions, the radius of gyration (Rg), the solvent accessible surface area (SASA), and other interaction plots like principal component analysis (PCA) and free energy landscape (FEL) plot were used to understand the stability of the complex.

## 3. Result

### 3.1. Homology Modeling

**Figure 1:**
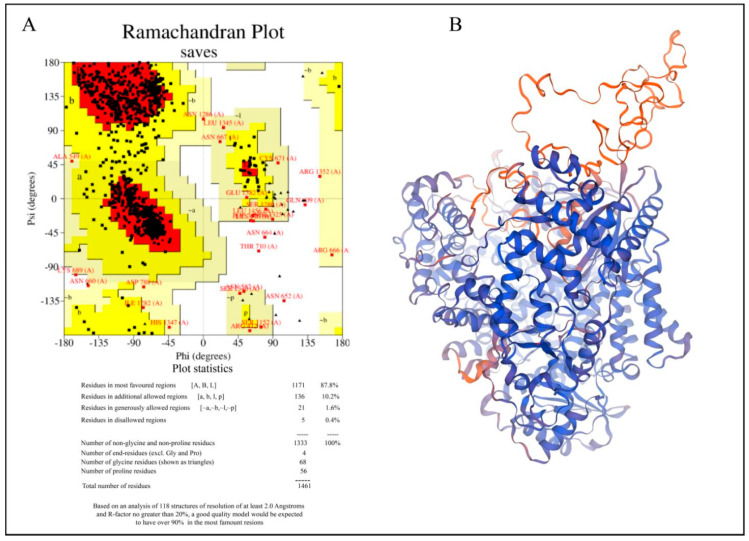
Homology−modeled structure of the Hendra virus RdRp protein. (**A**) Ramachandran plot of the modeled structure. (**B**) Three−dimensional ribbon representation of the homology model. Blue color represents regions with highest confidence and orange color denotes areas with lower confidence.

We used the Swiss Model for homology modeling because the RdRp of the Hendra virus does not have a crystal structure. An amino acid sequence from UniProt (O89344) is used as the input sequence. It then checks related structures in other databases, like the Protein Data Bank (PDB), and builds a 3D model using the best match template. Also, the tool employs scoring methods to evaluate the quality of the model [15]. A template with PDB ID 9BDQ, which depicts the structure of the replicating Nipah virus RNA polymerase complex-RNA-bound state, was used to build the target protein. It has an 86.72 sequence identity and a 0.52 Global Model Quality Estimation (GMQE). The Swiss Model-provided Ramachandran plot and the structure evaluation tools were used to verify the 3D model of the target protein. MD simulation was performed on the modeled structure in order to further relax the system. After MD simulation the three-dimensional (3D) structure of the RdRp protein of the Hendra virus exhibited significant structural stability and refinement. The average Cα deviation of 5.31 Å of the structure of the modeled protein over the 200 ns RMSD trajectory reveals that the RMSD is stabilized over the simulation. Based on the Ramachandran plot, 87.8% were located in the most favored regions; additionally, allowed regions were 10.2%, 1.6% in generously allowed regions, and only 0.4% of residues were found in disallowed regions; however, closer analysis revealed that these correspond to the protein’s loop regions. Compared to structural elements like α-helices and β-sheets, loop regions are naturally more flexible and less conformationally bound; thus, such variations are not unusual. Therefore, the overall quality or reliability of the protein model is not significantly compromised by the presence of these residues in the disallowed regions. The small percentage of residues in prohibited areas suggests that the model’s stereochemical quality is generally reliable, with only a few isolated variations that could be enhanced. Figure 1 represents the homology-modeled structure of the Hendra virus RdRp protein.

### 3.2. Virtual Screening Using Molecular Docking

**Figure 2:**
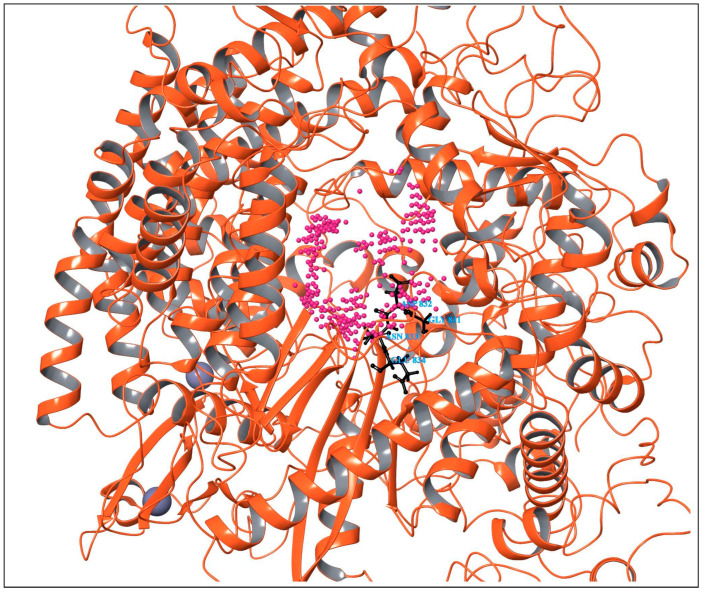
Predicted druggable sites in the RdRp of Hendra virus identified using the SiteMap module. The magenta entries represent the predicted binding pockets, with the GDNE motif within binding site 2. The black sticks represent its GDNE motif.

All possible druggable sites of the RdRp of the Hendra virus were mapped using SiteMap module in the Schrodinger suite. SiteMap generates a SiteScore that describes the binding site’s size, solvent exposure, tightness of interaction between site points and the receptor, balance between hydrophobicity and hydrophilicity, and degree of hydrogen bond between donation and acceptance [15]. A SiteScore greater than one is suggestive of a promising binding site. SiteMap analysis revealed the presence of six potential binding sites in the modeled protein. Among the predicted sites, binding sites 1, 2, and 3 had high site scores (1.077, 1.072, and 1.007), and their druggability scores (Dscores) were likewise high (1.062, 1.021, and 0.990), respectively. Among the predicted sites, the GDNE motif (GLY831, ASP832, ASN833, and GLU834) was located within the binding site 2, which showed a favorable SiteScore and Dscore. Thus, binding site 2 was chosen for molecular docking studies, based on the alignment of these key active site residues and the high druggability parameters. Table 1 and Supplementary Table S1 represents the scoring of all predicted binding sites. Figure 2 depicts the predicted druggable sites in the RdRp of the Hendra virus identified using the SiteMap module.

**Figure 3:**
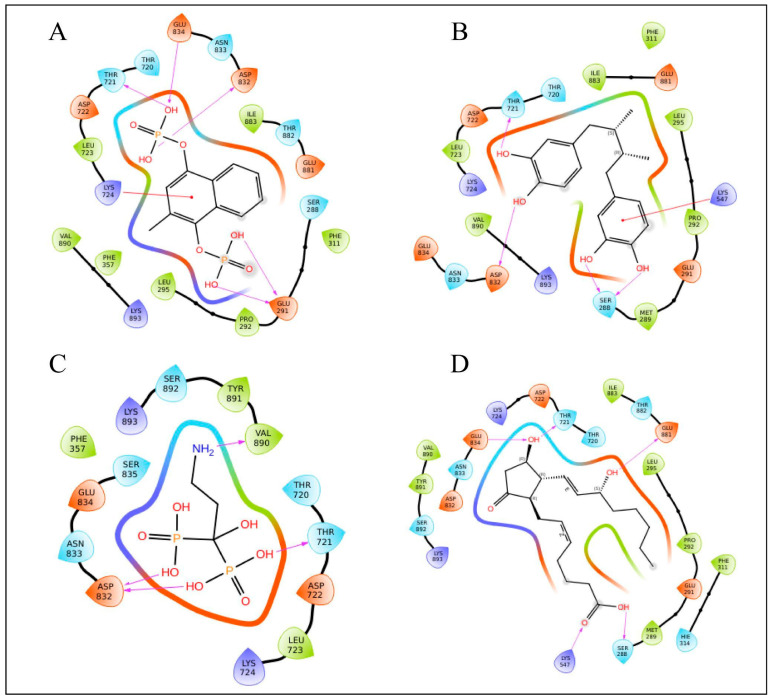
Ligand interaction diagram of RdRp of Hendra virus with FD approved drugs (**A**) Menadiol diphosphate, (**B**) Masoprocol, (**C**) Pamidronic acid, and (**D**) Dinoprostone.

The list of potential compounds was filtered based on their docking scores in comparison to standard drugs. Using the MM-GBSA binding free energy scores alongside the docking scores, which demonstrated effective stability between the target protein and specific therapeutic molecules, the top ligands were selected (Table 2). Menodiol diphosphate showed a binding free energy of −49.88 kcal/mol and a docking score of −8.417 kcal/mol. It stabilizes the drug–target interaction by forming hydrogen bonds with four amino acids: GLU834, THR721, ASP832, LYS724, and GLU291. Additionally, it forms a pi-cation interaction with LYS724. Pamidronic acid displayed a binding free energy of −34.29 kcal/mol and a docking score of −8.250 kcal/mol, forming four hydrogen bonds with VAL890, ASP832, and THR721. Masoprocol had a binding free energy of −39.69 kcal/mol and a docking score of −7.720 kcal/mol. This compound forms four hydrogen bonds with ASP832, THR721, and SER288, and there is a pi-cation interaction with LYS547. Dinoprostone showed a binding free energy of −46.90 kcal/mol and a docking score of −7.514 kcal/mol. It interacts with five amino acids, GLU834, THR721, GLU881, LYS547, and SER288, through hydrogen bonding. The RdRp domain is organized in a traditional right-hand “fingers-palm-thumb” pattern with seven catalytic motifs (A–G). The palm has motifs A–E, whilst the fingers have motifs F and G. The active site’s “GDNE” motif (residues 831–834) is found in motif C and targeting the conserved GDNE (831–834) motif of the RdRp domain, which forms the catalytic core of the polymerase, is particularly relevant for therapeutic interventions. All of the top-scoring compounds form hydrogen bonds with any of the residues in between 831 and 834, indicating a direct interaction with the active site. In addition to active site residues, THR721, ASP832, GLU291, LYS724, VAL890, SER288, LYS547, and GLU881 residues participate in hydrogen bonding. Interactions between repurposing candidate and RdRp of the Hendra virus are represented in Table 2 and Figure 3. The docking scores and the binding free energy for other identified drug candidates can be found in Supplementary Table S2.

### 3.3. ADMET Analysis

The absorption, distribution, metabolism, excretion, and toxicity (ADMET) properties of the top drug candidates were evaluated using AI Drug Lab [25]. These include molecular weight, oral bioavailability, blood–brain barrier (BBB) permeability, the number of hydrogen bond acceptors (HA), the number of hydrogen bond donors (HD), human intestinal absorption, lipophilicity, and plasma protein binding rate. Even though the selected compounds have FDA approval, not all approved drugs possess optimal ADMET properties, as these parameters are highly context-dependent and vary with the intended pharmacological site of action and therapeutic target. A compound approved for oncological or topical use may not necessarily exhibit ideal oral bioavailability or BBB permeability in a systemic antiviral context. To ensure target-specific pharmacokinetic suitability for the Hendra virus RdRp, we re-evaluated the ADMET parameters using AI Drug Lab [28]

**Figure 4:**
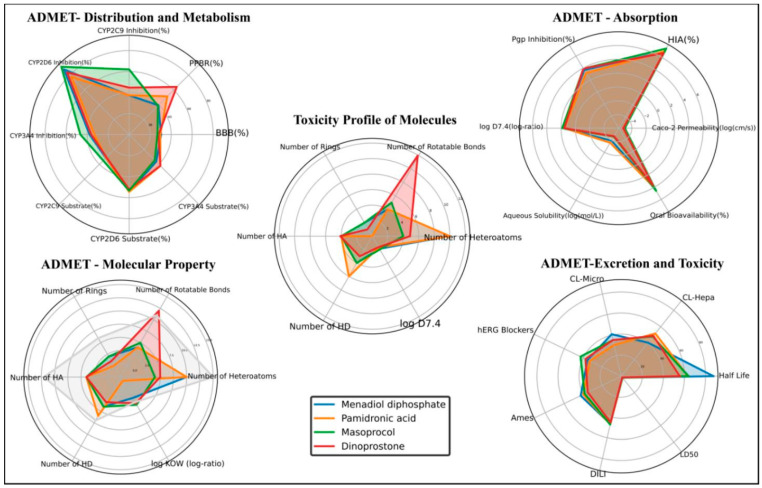
ADMET profiling of the top-scoring compounds.

All candidates meet the criteria of Lipinski’s Rule of Five. The most promising candidate was menadiol diphosphate, which had the longest half-life (87.5 h), good BBB penetration (30.48%), and intermediate oral bioavailability (41.71%). However, its substantial CYP2D6 inhibition also suggests potential metabolic liabilities. Masoprocol demonstrated the highest oral bioavailability at 45.13% and human intestinal absorption of 73.57%, suggesting excellent absorption. Overall, menadiol diphosphate emerged as the most balanced candidate overall, demonstrating acceptable toxicity and favorable pharmacokinetics. ADMET profiling of high-scoring compounds is depicted in Figure 4, Table 3, and Supplementary Table S3.

### 3.4. Molecular Dynamics

#### 3.4.1. Root Mean Square Deviation (RMSD)

**Figure 5:**
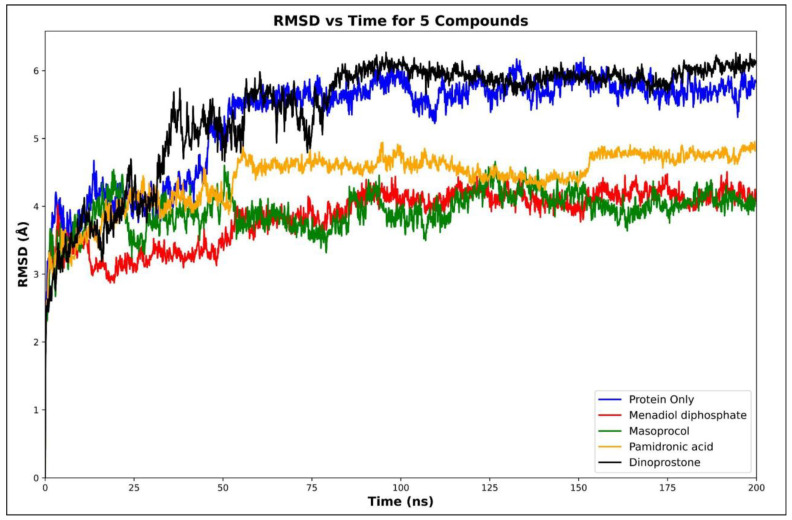
Cα RMSD plots of RdRp in complex with Menadiol diphosphate, Masoprocol, Pamidronic acid, Dinoprostone compared to the unbound (protein only) RdRp protein.

**Figure 6:**
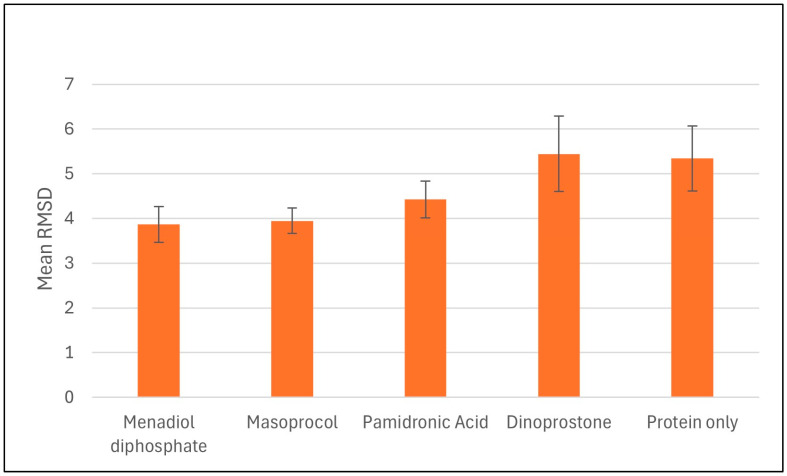
Average Cα RMSD values for RdRp and its ligand complexes during 200 ns MD simulations. Standard deviations represented by error bars, which indicate variations in structural stability between the systems.

Molecular dynamics (MD) simulations were performed on selected compounds based on their binding free energies, docking scores, and ADMET (Absorption, Distribution, Metabolism, Excretion, and Toxicity) properties. The compounds analyzed included dinoprostone, masoprocol, menadiol diphosphate, and pamidronic acid. To evaluate the conformational stability upon ligand binding, we conducted RMSD analyses for the protein only and its complexes with these ligands. Molecular dynamics simulations were carried out to assess the dynamics behavior and stability, and molecular docking analysis is performed to predict the binding affinities and interactions between ligands and targets. Molecular dynamics simulations were carried out to assess the dynamics behavior and stability Figure 5 represents the average protein Cα RMSD plots of RdRp in complex with menadiol diphosphate, masoprocol, pamidronic acid, and dinoprostone compared to the unbound (protein only) RdRp protein, while Figure 6 presents the mean RMSD values with standard deviation bars. According to Figure 5 all systems attained equilibrium after the initial 50–60 ns. The average Cα RMSD of the unbound RdRp was 5.31 ± 0.42 Å, whereas the ligand-bound complexes, menadiol diphosphate (3.86 ± 0.33 Å), masoprocol (3.94 ± 0.37 Å), pamidronic acid (4.42 ± 0.45 Å), and dinoprostone (5.43 ± 0.51 Å) show comparatively lower deviations. Together, these findings imply that masoprocol and menadiol diphosphate enhance the stability of the protein structure when compared to the protein alone. The most promising candidates were masoprocol and menadiol diphosphate due to their consistently low RMSD values.

#### 3.4.2. Root Mean Square Fluctuation (RMSF)

**Figure 7:**
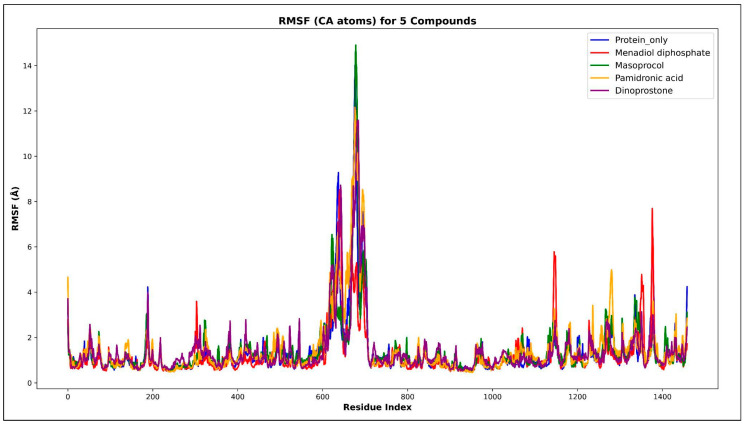
Illustrates the RMSF value of Cα throughout the 200 ns simulation period. The blue color represents protein only, the red color represents the Menadiol diphosphate–Protein complex, the orange color represents the Pamidronic acid–Protein complex, the green color represents the Masoprocol–Protein complex, and the violet color represents the Dinoprostone–Protein complex.

**Figure 8:**
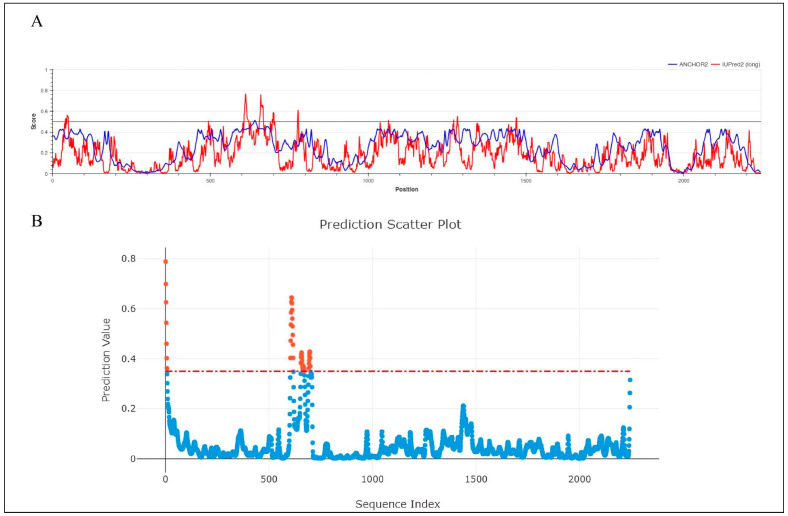
Prediction of intrinsically disordered regions (IDRs) in the Hendra virus RdRp protein. (**A**) IUPred2A disorder prediction profile showing residue-wise disorder probability across the RdRp sequence. The red line represents the IUPred2A score, and the blue line corresponds to ANCHOR2 predictions, indicating potential binding regions within disordered segments. Residues with scores above 0.5 are considered disordered. Orange dots represent positive predicted site(residues with prediction scores above the threshold) and blue dots represent negative predicted site(residues with prediction scores below the threshold). (**B**) PUNCH prediction scatter plot illustrating residue-level disorder propensity. The threshold of 0.4 for disorder classification is indicated by the red dashed line.

The average displacement of each atom from its mean position during a molecular dynamics (MD) simulation is quantified by the root mean square fluctuation (RMSF). Areas with higher RMSF values indicate greater mobility, while lower values suggest reduced mobility. Figure 7 represents the RMSF value of Cα throughout the 200 ns simulation period. According to Figure 7, the average RMSF value for the unbound RdRp was 1.3198 Å, but the ligand-bound complexes, menadiol diphosphate (1.2126 Å), masoprocol (1.3672 Å), pamidronic acid (1.4514 Å), and dinoprostone (1.3997 Å) showed slightly smaller fluctuations. In Figure 8, higher fluctuations were observed in the residue range 621–721, which corresponds to a disordered region found by IUPred2A [25], PUNCH [26], and ANCHOR2 [29]. From the analysis it was observed that amino acids in the range 605–705 belong to the protein’s disordered region. These areas clearly lack a defined secondary structure. Consequently, the existence of disordered regions exhibits greater fluctuation and is expected in those regions thus justifying the observance of higher RMSFrmsf values in those regions. In order to quantify fluctuations in the intrinsically disordered region (residues 605–705), each protein–ligand complex’s average RMSF values were calculated and compared with the protein only. The results (Supplementary Table S4) show that the Menadiol diphosphate complex exhibited the lowest average RMSF (3.585 Å ± 1.721 Å). During molecular dynamics (MD) simulation, low average RMSF (Root Mean Square Fluctuation) values in the disorder regions of the menadiol diphosphate protein complex show increased structural stability and decreased flexibility in those regions. This implies that with drug binding, the normally flexible disorder regions may become more ordered or stabilized, reflecting limited atomic position deviations throughout the simulation [29].

#### 3.4.3. Protein–Ligand Interaction

**Figure 9:**
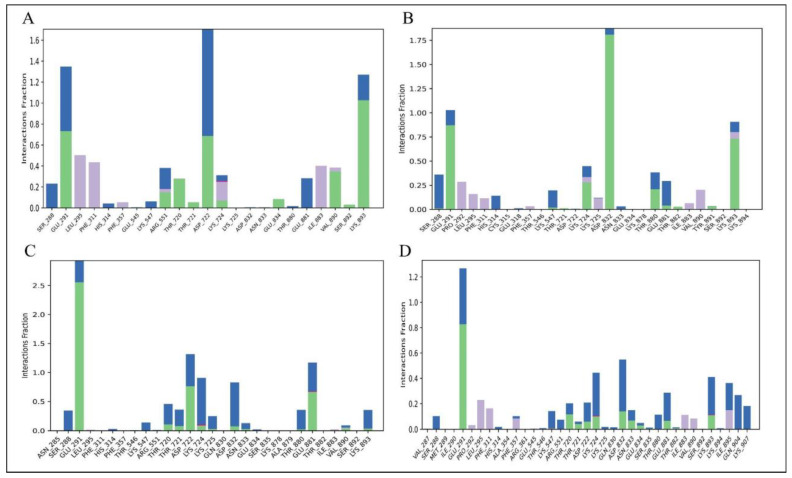
Protein–Ligand contact between (**A**) Menadiol diphosphate, (**B**) Masoprocol, (**C**) Pamidronic acid, and (**D**) Dinoprostone. Different interaction types are represented by color coded bars: hydrogen bonds (green), hydrophobic interactions (purple), ionic interactions (red) and water bridges (blue).

Electrostatic forces, hydrophobic interactions, and hydrogen bonds were among the stabilizing contacts found in the analysis of the Hendra virus RdRp protein and ligand interactions. Hydrogen bonds are particularly important for maintaining protein stability and enhancing ligand binding at the active site. The interaction between ligands and proteins is depicted in Figure 9, while detailed 2D interaction profiles are shown in Supplementary Figure S1. Menadiol diphosphate demonstrated hydrogen bonding with the important active site residue GLU834 in addition to LYS893, ASP722, and GLU291. Masoprocol provides key supporting interactions by involving the strong hydrogen bond with ASP832, along with LYS893 and GLU291. Interactions between pamidronic acid and residues GLU291, ASP722, GLU881, and with the active site residues ASP832 and ASN833 were demonstrated by hydrogen bonds. Dinoprostone also showed substantial interactions with GLU291 as well as significant binding at the active site via hydrogen bonds with ASP832, ASN833, and GLU834. These findings highlight the significance of hydrophobic and electrostatic interactions along with the broad network of hydrogen bonds. Together, these interactions stabilize the ligand–protein complex within the RdRp active site, potentially increasing the binding affinity and inhibitory potency of these compounds.

#### 3.4.4. Solvent Accessible Surface Area (SASA)

**Figure 10:**
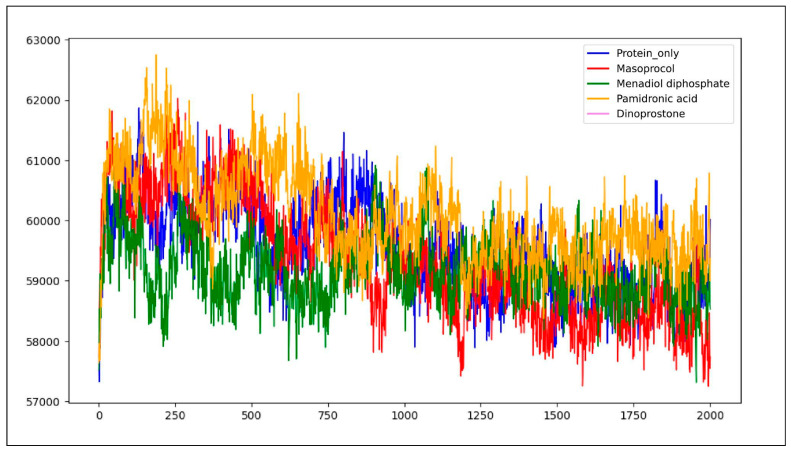
Solvent accessible surface area (SASA) graph plotting the four ligand–protein complexes. The blue color represents protein only, the green color indicates the Menadiol diphosphate–Protein complex, orange color represents the pamidronic acid–Protein complex, the red color indicates the Masoprocol–Protein complex, and the purple color represents the Dinoprostone–Protein complex.

Solvent accessible surface area (SASA) analysis quantifies the extent of a molecule’s surface that remains exposed and accessible to a solvent molecule during a 200 ns molecular dynamics (MD) simulation. The total area of a protein that is accessible for solvent molecules is measured by SASA. Figure 10 represents a solvent accessible surface area (SASA) graph plotting the four complexes. The protein only had an average SASA of 59,588.3 Å^2^, indicating a higher level of residues exposed to solvents. A protein with a lower SASA score has less water exposure because its hydrophobic portions are folded inward. On the other hand, a higher SASA value indicates that solvent molecules can reach more hydrophobic regions, which implies reduced compactness and potential destabilization of the protein–ligand complex [30].

Average SASA value of protein only indicates a higher level of residues exposed to solvents, whereas Menadiol diphosphate has an average SASA value of 59,175.8 Å^2^. Average SASA value for masoprocol, pamidronic acid, and dinoprostone is 60,082.1 Å^2^, 59,355 Å^2^ and 59,749.5 Å^2^, respectively. The reduction in SASA after ligand binding indicates that these substances encourage a more compact structural conformation by decreasing water accessibility and isolating hydrophobic regions. Ligand has occupied the volume of the pocket more, thus reducing the solvent accessible surface area. Ligand interactions generally decreased solvent accessibility in comparison with protein only, according to SASA analysis, indicating a tendency for more compact structural conformations. The most significant reduction in SASA across the complexes was exhibited in menadiol diphosphate, suggesting enhanced stability due to the decreased water exposure. With acceptable toxicity and good pharmacokinetics, menadiol diphosphate was found to be the most balanced candidate, despite its potential for CYP-mediated interactions.

#### 3.4.5. Radius of Gy Ration (Rg)

The mass of atoms within the center of mass of protein is indicated by the radius of gyration (Rg) [31]. The Rg value indicates the structural compactness required for the conformational stability of proteins, while a lower Rg number suggests that the protein is not experiencing major structural changes and the protein remains stable and maintains its stable native form, whereas a higher Rg value indicates reduced compactness and possible expansion of the protein structure.

**Figure 11:**
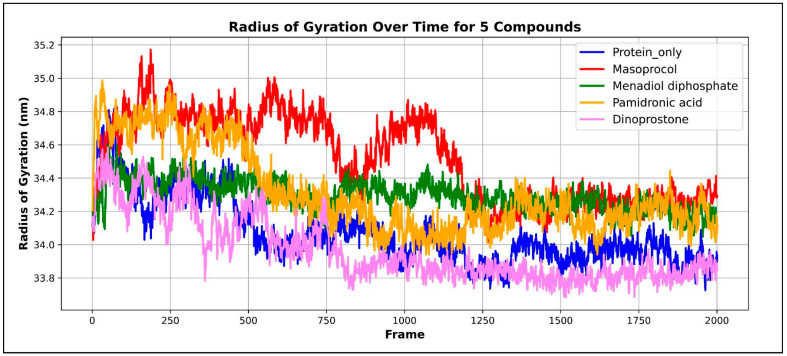
Radius of gyration (Rg), a graph plotting the four complexes. Protein only is represented by the color blue; the green color represents the Menadiol diphosphate−Protein complex; Pamidronic acid−Protein complex is represented by the orange color; the red color represents the Masoprocol−Protein complex; and Dinoprostone−Protein complex by the color purple.

Figure 11 depicts a radius of gyration (Rg) graph plotting the four complexes. The unbound protein has an average Rg value of 34.0685 Å ± 0.1953 Å, whereas menadiol diphosphate shows an average Rg value of 34.295 Å ± 0.0912 Å. For masoprocol, pamidronic acid, and dinoprostone, the average Rg values are 34.5118 Å ± 0.2577 Å, 34.3141 Å ± 0.2507 Å, and 33.9683 Å ± 0.1983 Å, respectively. This range of values implies that there was minimal protein rearrangement, resulting in significant stability. Overall structural stability was indicated by the Rg analysis, which showed that all protein–ligand complexes maintained values close to the unbound protein over the simulation. Menadiol diphosphate, masoprocol, and pamidronic acid showed somewhat higher Rg values, indicating moderate reductions in compactness, but dinoprostone showed slightly lower Rg value, indicating greater compactness.

#### 3.4.6. The Binding Free Energy of Post-Molecular Dynamics

The binding free energy of protein–ligand complex frames extracted from the MD trajectory was evaluated using the MM-GBSA method following the molecular dynamics (MD) run. From the average binding free energy obtained, the standard deviations were computed. Interestingly, menadiol diphosphate had the highest binding free energy, measuring −60.87 ± 5.68 kcal/mol, while the binding free energy measured before the MD run was −49.88 kcal/mol. Following the MD simulation, the binding free energy for masoprocol improved from −39.69 kcal/mol to −48.61 ± 4.61 kcal/mol. Prior to the MD run, the binding free energy of pamidronic acid was −34.29 kcal/mol; it subsequently dropped to −18.70 ± 7.52 kcal/mol. The binding free energy of dinoprostone is −42.13 ± 5.90 kcal/mol, which is lower than the binding free energy of −46.90 kcal/mol prior to the MD run. The binding free energy along with contributing energies of the lead candidates are represented in Table 4. After conducting MD simulations, the MM-GBSA analysis indicated that menadiol diphosphate exhibited the most favorable binding pose with a free energy of −65.59 ± 5.29 kcal/mol, representing a significant improvement over its pre-MD MM-GBSA value. The binding energy calculations after MD simulations indicated that masoprocol and menadiol diphosphate exhibited improved binding affinities, demonstrating stable and favorable interactions with the target protein. In contrast, pamidronic acid and dinoprostone exhibited reduced binding stability, suggesting that they showed higher structural fluctuations and weaker interactions with the protein during the simulation and were less energetically favorable.

#### 3.4.7. Principal Component Analysis (PCA)

**Figure 12:**
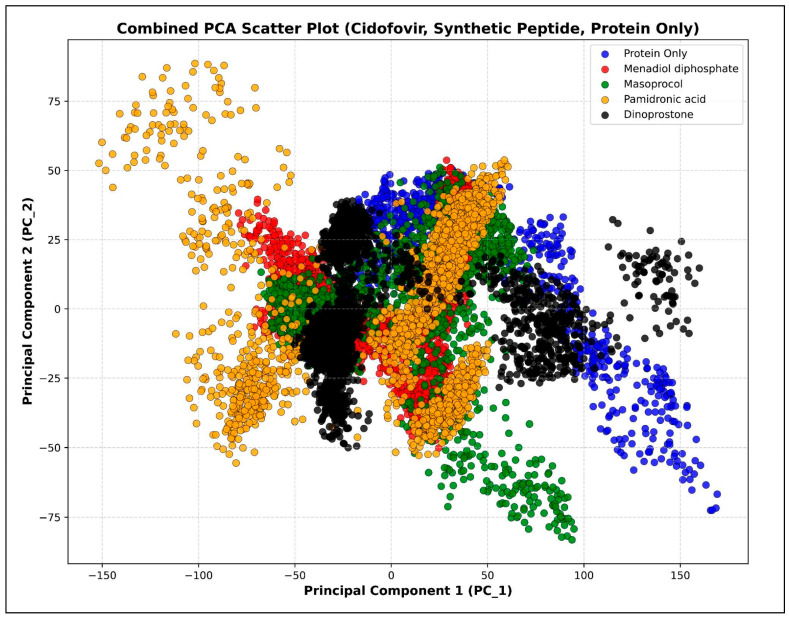
Principal Component Analysis (PCA) of protein only in blue color, menadiol diphosphate–protein complex in red color, masoprocol–protein complex in green color, pamidronic acid–protein complex in yellow color, and dinoprostone–protein complex in black color.

Principal Component Analysis (PCA) is a statistical technique used to rank observed motions from the largest to the smallest geographic scales, particularly when applied to multivariate data. This method effectively reduces the dimensions needed to describe protein dynamics [32]. The protein behavior changes before and after binding are depicted as a scatter plot in Figure 12. Among the complexes, menadiol diphosphate showed the most compact clustering of conformations, indicating good stability and minimal structural variations. The protein only exhibited greater inherent flexibility by exploring a wider structural space. Therefore, these findings support the idea that, when menadiol diphosphate is docked with the protein, this ligand interaction stabilizes protein structure and restricts large-scale conformational variations.

#### 3.4.8. Free Energy Landscape (FEL)

To understand the energy changes during conformational changes in receptor–ligand interactions, it is important to examine the Gibbs free energy landscape. According to classical thermodynamics, the complex structure formed by the interaction of a protein and a ligand should correspond to the conformation that exhibits the lowest binding free energy. This interaction is representative of a systematic thermodynamic equilibrium.

**Figure 13:**
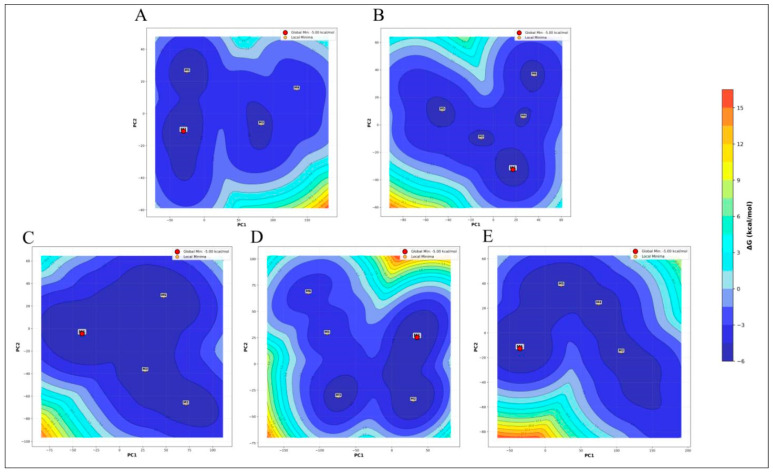
Free Energy Landscape (FEL) of (**A**) Protein only, (**B**) Menadiol diphosphate-protein complex, (**C**) Masoprocol-Protein complex, (**D**) Pamidronic acid-Protein complex, and (**E**) Dinoprostone-protein complex.

It is crucial to understand the kinetic and thermodynamic properties that influence the behavior of complex biomolecular systems. An energetically favorable contact that promotes the creation of a stable complex between the ligands and proteins is indicated by a low Gibbs free energy [33]. Comparative free energy landscape (FEL) analysis of protein alone, menadiol diphosphate, masoprocol, pamidronic acid, and dinoprostone following molecular dynamics simulation shows significant conformational changes (Figure 13). While all systems exhibit the global minima at −5.00 kcal/mol, indicating similar thermodynamic stability in their lowest energy state, their landscape topologies offer important insights into the adaptability, flexibility, and possible binding behavior. Masoprocol and pamidronic acid, the compounds with the lowest FELs, have three to four local minima in addition to the global minima. These wide shallow basins imply limited conformational sampling and fewer microstates, which may indicate less structural flexibility. In pamidronic acid and dinoprostone, the frequency of local minima and contour separation show a moderately increased complexity, suggesting improved conformational variety and the existence of distinct metastable states.

In contrast, the Menadiol diphosphate system is notable for having a high landscape complexity. The five distinct minima (M1, M3, M4, M5, and M6) are dispersed among distinct energy basin segments. Compared to other systems, the contour lines display a greater separation between these minima and higher energy barriers, suggesting that each conformational state is very persistent. Because of its enhanced stability and diversity, menadiol diphosphate can bind to a variety of stable sites and respond dynamically to its environment.

All of these findings indicate menadiol diphosphate having the most stable and diverse conformational landscape. In addition to offering more chances for successful and targeted binding, this could also be linked to increased functional adaptability in a biological context. Dinoprostone and pamidronic acid hold an intermediate position, while protein and masoprocol only provide more limited, less dynamic landscapes.

Thus, FEL analysis based directly on the observed contour plots clearly supports menadiol diphosphate as the most conformationally versatile and adaptable system, given its broader distribution and separation of stable energy states.

## 4. Discussion

In Australia, the Hendra virus represents a significant zoonotic illness with a high prevalence in flying fox reservoirs and detrimental effects on both human and equine health with a high fatality rate [34]. It falls under the same classification as the Ebola virus, biosafety category 4, which is the highest level of biocontainment [35]. This study employed virtual screening, molecular docking, and molecular dynamics (MD) simulations to discover potential inhibitors of the Hendra virus (HeV) RNA-dependent RNA polymerase (RdRp). As the RNA-dependent RNA polymerase (RdRp) is crucial for both transcription and viral replication, it serves as an important target for therapeutic intervention, especially in the absence of approved antivirals or vaccines to prevent human infections caused by the Hendra virus (HeV). This study suggests a method for repurposing current drug-like molecules for accelerating antiviral discovery against emerging zoonotic infections.

Based on the preliminary docking results and the MM-GBSA studies, menadiol diphosphate, masoprocol, pamidronic acid, and dinoprostone were identified as potential candidates for RdRp binding. Menadiol diphosphate consistently showed the strongest hydrogen bonding at the active site residues (831–834) as well as the most favorable binding free energy both before and after molecular dynamics simulations. Furthermore, masoprocol demonstrated an improved binding affinity and more durable hydrogen bond formation in MD simulations, suggesting beneficial dynamic interactions. After the MD simulations, lower binding free energies for pamidronic acid and dinoprostone were identified, indicating less stable protein–ligand complexes. The MD-based structural studies provided more insight into the stabilizing effects of these ligands on RdRp.

However, upon initial binding, dinoprostone and pamidronic acid produced more conformational heterogeneity and protein changes. The RMSD and RMSF results showed that the complexes of menadiol diphosphate and masoprocol exhibited structural stability with minor alterations. These findings were confirmed by studies using SASA and Rg, which demonstrated that menadiol diphosphate in particular reduced solvent accessibility and made proteins more compact. The findings indicate that certain ligands can enhance the structural stability of RdRp complex and binding effectiveness, two critical components of antiviral action. Further details regarding the conformational dynamics that ligands induce have been discovered through the use of PCA. Correlated motions were stabilized and compact structure clustering was promoted by masoprocol and menadiol diphosphate, indicating beneficial cooperative dynamics. However, complexes of dinoprostone and pamidronic acid showed increased or extensively distributed anti-correlated motions, which are consistent with structural effects that produce instability over extended distances. When assessing potential antiviral drugs, our findings highlight the importance of considering protein flexibility and long-range conformational effects in addition to binding affinities. As RdRp inhibitors for the Hendra virus, our results indicate that masoprocol and menadiol diphosphate are promising candidates for future research and development. Their potential efficacy is enhanced by their capacity to maintain favorable binding energies, promote compact conformational states, and stabilize the active site. Through experimental confirmation, such as biochemical RdRp inhibition studies and in vitro antiviral testing in HeV-infected systems, the therapeutic value of these findings has to be confirmed.

In conclusion, our comprehensive computational method identified masoprocol and menadiol diphosphate as the most potential inhibitors of Hendra virus RdRp. These results show the effectiveness of MD-based dynamic analysis and in silico drug repurposing in accelerating the development of novel treatments for zoonotic illnesses, while also adding to the growing field of HeV antiviral research.

## 5. Conclusions

A thorough computational investigation of possible RdRp inhibitors is presented in this study in order to fight the Hendra virus, a highly lethal zoonotic illness for which there are currently no effective antiviral therapies. The most promising therapeutic candidates are identified as masoprocol and menadiol diphosphate through the use of molecular dynamics simulations, ADMET analysis, and virtual screening. The two compounds demonstrated the ability to maintain compact and stable RdRp conformations, strong hydrogen bond networks, significant binding affinities, and favorable free energy profiles. However, the dynamic stability of dinoprostone and pamidronic acid was reduced, suggesting a limited ability for long-term inhibition. The findings suggest that menadiol diphosphate and masoprocol are promising drug candidates for therapeutic development. However, in vitro and in vivo research is still needed to confirm their therapeutic value and efficacy.

## Figures and Tables

**Table 1:** Scoring of binding sites from Sitemap.

Sl.No	Site Number	SiteScore	Dscore
1	Binding_site_1	1.077	1.062
2	Binding_site_2	1.072	1.021
3	Binding_site_3	1.007	0.990
4	Binding_site_4	1.007	0.988
5	Binding_site_5	1.004	0.996

**Table 2:** Docking score and binding free energy of the selected drugs.

Sl.No.	Compound Name	Binding Free Energy kcal/mol	Docking Score kcal/mol	Number of Hydrogen Bonds	Interacting Residues
1	Menodiol diphosphate	−49.88 kcal/mol	−8.417 kcal/mol	5	GLU834, THR721, ASP832, GLU291, LYS724
2	Masoprocol	−39.69 kcal/mol	−7.720 kcal/mol	4	ASP832, THR721, SER288, LYS547
3	Pamidronic acid	−34.29 kcal/mol	−8.250 kcal/mol	4	VAL890, ASP832, THR721
4	Dinoprostone	−46.90 kcal/mol	−7.514 kcal/mol	5	GLU834, THR721, GLU881, LYS547, SER288

**Table 3:** ADMET Profiling of high-scoring compounds.

Properties	Menadiol Diphosphate	Pamidronic Acid	Masoprocol	Dinoprostone
Molecular Weight	334.0	235.0	302.15	352.22
Number of Heteroatoms	10	10	4	5
Number of Rotatable Bonds	4	4	5	12
Number of Rings	2	0	2	1
Number of HA	4	4	4	4
Number of HD	4	6	4	3
log KOW	2.09	−1.66	3.57	3.25
Caco-2 Permeability	−5.3	−5.23	−5.11	−5.33
HIA	68.36	68.9	73.57	65.91
Pgp Inhibition	37.76	32.46	39.93	40.0
log D7.4	1.88	1.68	1.98	1.69
Aqueous Solubility	−3.88	−3.53	−4.54	−4.62
Oral Bioavailability	41.71	39.96	45.13	35.92
BBB	30.48	29.7	26.98	27.98
PPBR	39.45	51.44	38.8	64.14
VDss	2.82	2.48	3.36	3.0
CYP2C9 Inhibition	37.31	37.43	62.03	44.7
CYP2D6 Inhibition	87.76	77.95	91.3	83.64
CYP3A4 Inhibition	37.61	33.04	46.44	36.09
CYP2C9 Substrate	31.46	31.19	34.93	30.51
CYP2D6 Substrate	54.28	54.55	53.5	52.21
CYP3A4 Substrate	36.51	42.14	34.54	42.06
Half Life	87.5	63.47	63.9	55.55
CL-Hepa	40.75	51.89	48.08	48.94
CL-Micro	40.86	30.47	35.28	35.22
hERG Blockers	35.52	33.18	42.92	37.66
Ames	42.75	40.07	38.62	35.32
DILI	46.65	47.1	47.13	45.07
LD50	2.01	1.57	2.01	1.46

**Table 4:** The binding free energy along with contributing energies of the lead candidates.

Drug Leads	Binding Free Energy (kcal/mol)	Van Der Waals Energy (kcal/mol)	Coulomb Energy (kcal/mol)	Solv GB (kcal/mol)	Lipophilic Energy (kcal/mol)	H Bond (kcal/mol)
Menadiol diphosphate	−58.99 ± 4.44	−40.68 ± 2.25	−35.23 ± 4.34	30.83 ± 1.94	−12.72 ± 0.80	−3.06 ± 0.47
Masoprocol	−48.61 ± 4.61	−38.73 ± 3.12	−18.99 ± 3.64	29.56± 2.08	−18.73± 1.37	−3.20 ± 0.45
Dinoprostone	−42.13 ± 5.90	−43.25 ± 3.20	−12.75 ± 9.42	29.69 ± 6.65	−15.51 ± 1.63	−1.35 ± 0.61
Pamidronic acid	−18.70 ± 7.52	−15.85 ± 5.80	−31.15 ± 10.94	32.56 ± 6.17	−1.77 ± 0.82	−3.78 ± 1.01

## Data Availability

The data supporting the findings of this study are available from the corresponding author upon request.

## Supplementary Materials

The following supporting information can be downloaded at https://www.mdpi.com/article/10.3390/v17121613/s1, Supplementary Table S1: Represents the scoring of all predicted binding sites, Supplementary Table S2: docking scores and the binding free energy for all identified drug candidates, Supplementary Table S3: ADMET profiling of high-scoring compound, Supplementary Table S4: Average RmSF values of the intrinsically disorded region (residues 605–705), Supplementary Figure S1: 2D interaction profiles beteween protein and ligands.
