# Supplementary material for: Repurposing FDA-Approved Drugs as Hendra Virus RNA-Dependent RNA Polymerase Inhibitors: A Comprehensive Computational Drug Discovery Approach
Source: Viruses. 2025 Dec 13;17(12):1613. doi: 10.3390/v17121613 (PMC12737397; doi:10.3390/v17121613)
Supplement: Supplementary file 1 [file viruses-17-01613-s001.zip › Supplemetary Figure S1.pdf]

Supplementary Figure S1

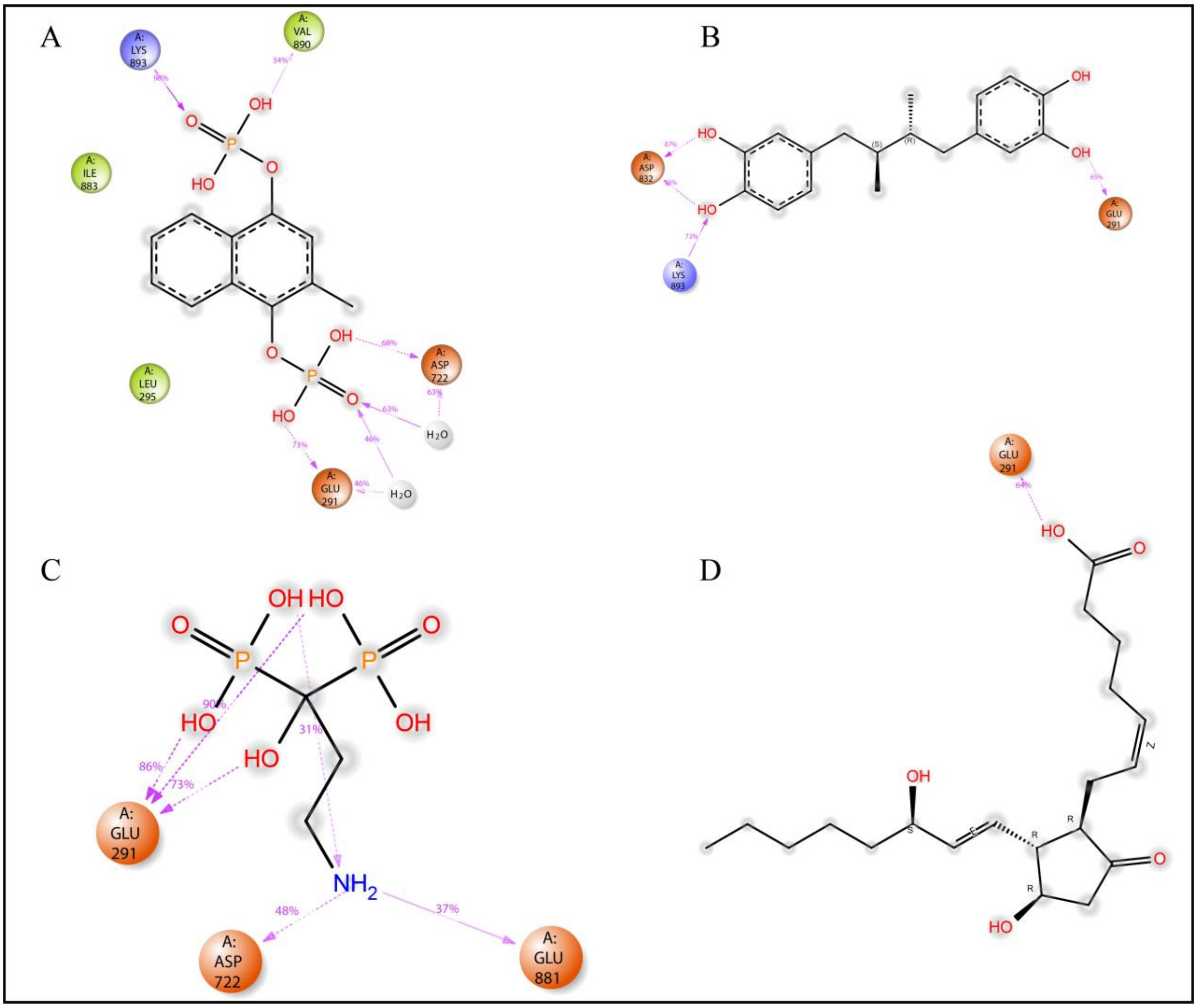

Supplementary Figure S1. Two-dimensional (2D) protein-ligand interaction diagrams of the Hendra virus RdRp active site with (A) menadiol diphosphate, (B) masoprocol, (C) pamidronic acid, and (D) dinoprostone
