# Supplementary material for: Repurposing FDA-Approved Drugs as Hendra Virus RNA-Dependent RNA Polymerase Inhibitors: A Comprehensive Computational Drug Discovery Approach
Source: Viruses. 2025 Dec 13;17(12):1613. doi: 10.3390/v17121613 (PMC12737397; doi:10.3390/v17121613)
Supplement: Supplementary file 1 [file viruses-17-01613-s001.zip › Supplemetary Table_Hendra.pdf]

Supplementary Table S1

Sl.No	Site number	SiteScore	Dscore
1	Binding_site_1	1.077	1.062
2	Binding_site_2	1.072	1.021
3	Binding_site_3	1.007	0.990
4	Binding_site_4	1.007	0.988
5	Binding_site_5	1.004	0.996

Supplementary Table S2

FDA approved drugs	Docking Score	MMGBSA DG Bind
Acipimox	-9.242	-42.21
Imidurea	-9.04	-30.12
Menadiol diphosphate	-8.417	-49.88
Pamidronic acid	-8.25	-34.29
Risedronic acid	-8.205	-38.86
Technetium Tc-99m medronate	-8.153	-19.85
Dapagliflozin	-8.144	-36.33
Oteracil	-8.112	-31.38
Tiludronic acid	-7.886	-42.34
Enprofylline	-7.809	-20.99
Pyridoxal phosphate	-7.798	-46.7
Zoledronic acid	-7.732	-41.4
Masoprocol	-7.72	-39.69
Fenoterol	-7.697	-38.59
Arformoterol	-7.663	-47.26
Dinoprostone	-7.514	-46.9
Arbutin	-7.484	-26.17
Etidronic acid	-7.418	-35.21
Kappadione	-7.349	-45.06
Alcloxa	-7.335	-30.03
Alvimopan	-7.219	-45.77
Bimatoprost	-7.189	-44.42
Alendronic acid	-7.188	-33.77
Cefradine	-7.121	-37.83
Arbutamine	-7.021	-39.08
Gluconolactone	-7.02	-35.36
Fructose	-6.943	-20.29
Loracarbef	-6.927	-41.37
Formoterol	-6.916	-35.09
Amoxicillin	-6.895	-31.14
Enalaprilat	-6.849	-18.94
Clodronic acid	-6.806	-37.77
Mitoxantrone	-6.772	-42.94
Olsalazine	-6.743	-32.19
Trifluridine	-6.74	-33.09
Calcium phosphate dihydrate	-6.713	-31.35
Sodium phosphate, dibasic	-6.7	-31.34
Calcium Phosphate	-6.7	-31.34
Sodium phosphate, monobasic	-6.7	-31.34
Dipotassium phosphate	-6.7	-31.34
Phosphoric acid	-6.699	-31.34

Supplementary Table S3

Properties	Value	Unit	Menadiol diphosphate	Pamidronic acid	Masoprocol	Dinoprostone
Molecular Weight	Optimal:100 - 600	kg/mol	334	235	302.15	352.22
Number of Heteroatoms	Optimal: 1-15	/	10	10	4	5
Number of Rotatable Bonds	Optimal:<= 11	/	4	4	5	12
Number of Rings	Optimal:<= 6	/	2	0	2	1
Number of HA	Optimal:<= 12	/	4	4	4	4
Number of HD	Optimal:<= 7	/	4	6	4	3
log KOW	Optimal: 0-3	log-ratio	2.09	-1.66	3.57	3.25
Caco-2 Permeability	Optimal: >-5.15	log(cm/s)	-5.3	-5.23	-5.11	-5.33
HIA	Poor:<=30% , Medium: 30%-80%, Optimum: >=80%	%	68.36	68.9	73.57	65.91
Pgp Inhibition	Poor:<=30% , Medium: 30%-70%, Optimum: >=70%	%	37.76	32.46	39.93	40
log D7.4	Optimal: 1-3	log-ratio	1.88	1.68	1.98	1.69
Aqueous Solubility	Soluble : -2 – 0, Slightly soluble: -4 - -2, Insoluble: < -4	log(mol/L)	-3.88	-3.53	-4.54	-4.62
Oral Bioavailability		%	41.71	39.96	45.13	35.92
BBB	Optimal:<=30% , Medium: 30%-70%,Poor: >=70%	%	30.48	29.7	26.98	27.98
PPBR	Optimal:<=90% ,Poor: >90%	%	39.45	51.44	38.8	64.14
VDss	Optimal: 0.04-20, poor: Otherwise	L/kg	2.82	2.48	3.36	3
CYP2C9 Inhibition		%	37.31	37.43	62.03	44.7
CYP2D6 Inhibition		%	87.76	77.95	91.3	83.64
CYP3A4 Inhibition		%	37.61	33.04	46.44	36.09
CYP2C9 Substrate		%	31.46	31.19	34.93	30.51
CYP2D6 Substrate		%	54.28	54.55	53.5	52.21
CYP3A4 Substrate		%	36.51	42.14	34.54	42.06
Half Life		hr	87.5	63.47	63.9	55.55

CL-Hepa		uL min-1 (106 cells)-1	40.75	51.89	48.08	48.94
CL-Micro		mL min-1 g-1	40.86	30.47	35.28	35.22
hERG Blockers	Poor:<=30% , Medium: 30%-70%, Optimum: >=70%	%	35.52	33.18	42.92	37.66
Ames	Poor:<=30% , Medium: 30%-70%, Optimum: >=70%	%	42.75	40.07	38.62	35.32
DILI	Poor:<=30% , Medium: 30%-70%, Optimum: >=70%	%	46.65	47.1	47.13	45.07
LD50		-log(mol/kg)	2.01	1.57	2.01	1.46

Supplementary Table S4

**Compounds**	**Average**	**Standard deviation**
Menadiol diphosphate	3.585	1.721
Masoprocol	4.335	3.079
Pamidronic acid	5.292	2.659
Dinoprostone	4.788	2.805
Protein_only	4.539	2.903
